# Implementing Large-Scale Data-Driven Quality Improvement in Assisted Living

**DOI:** 10.1016/j.jamda.2021.12.025

**Published:** 2021-12-31

**Authors:** Edmond Ramly, Reid Parks, Theresa Fishler, James H. Ford, David Zimmerman, Susan Nordman-Oliveira

**Affiliations:** a Department of Family Medicine and Community Health, School of Medicine and Public Health, University of Wisconsin–Madison, Madison, WI, USA; b Department of Industrial and Systems Engineering, University of Wisconsin–Madison, Madison, WI, USA; c Department of Medicine, School of Medicine and Public Health, University of Wisconsin–Madison, Madison, WI, USA; d School of Pharmacy – Social and Administrative Sciences Division, University of Wisconsin–Madison, Madison, WI, USA

**Keywords:** Assisted living, quality improvement, implementation

## Abstract

**Objectives:**

Develop and evaluate the implementation of a proposed model for large-scale data-driven quality improvement in assisted living.

**Design:**

We conducted a mixed-methods evaluation of the implementation of a large-scale data-driven quality improvement collaborative of Wisconsin assisted living communities (ALCs).

**Setting and Participants:**

The model has been voluntarily implemented by 810 Wisconsin-licensed ALCs serving >20,000 residents.

**Methods:**

The model was codesigned iteratively 2009–2012 by a public-private multistakeholder advisory group. Using system usage statistics and project records, we evaluated implementation outcomes: appropriateness, acceptability, adoption, feasibility, fidelity, penetration, and sustainability.

**Results:**

Implementation for ≥1 quarter was feasible for 92% of the 810 ALCs that enrolled. The model has been deemed appropriate and acceptable by public-private stakeholders representing residents, providers, regulators, and payers, and appropriateness for ALCs serving different populations has been iteratively improved through targeted workgroups. The model is currently adopted in Wisconsin by 31% of the 1573 ALCs in provider associations. Among adopters, 88% on average implemented the model with fidelity to key membership rules per quarter. The model achieved demographic and institutional penetration by currently reaching 24% of Wisconsin ALC residents and by leveraging initial grant funding to become integrated in Wisconsin’s annual Medicaid budget and being central to Wisconsin’s incentive program to managed care organizations. Model implementation for 8 years has been sustained by member enrollment for nearly 4 years on average, with 71% of members enrolled >2 years and sustained early adopters representing 37% that have been enrolled >5 years.

**Conclusions and Implications:**

This is the first implementation study of large-scale data-driven quality improvement in assisted living, despite its demonstrated value in other health care sectors. The article proposes a model with core components and implementation strategies drawing on a decade-long public-private collaboration. The implementation study findings establish a promising path and future directions for wider implementation.

The continued growth and need for assisted living communities (ALCs) raises concerns about the quality of care received by their residents.^1,2^ ALCs now serve populations with health needs that are increasingly similar in complexity and severity to those served by nursing homes.^3,4^ However, unlike nursing homes, quality in ALCs is subject to limited requirements, oversight, enforcement, resources, and clarity.^5^ These limitations include absence of federal oversight,^6^ state regulatory oversight with limited and variable stringency,^7,8^ limited resources and consistency in state monitoring and enforcement,^9^ limited ombudsmen resources,^10^ and lack of clarity in governance structures.^11^ As a result, quality issues in assisted living persist.^12^

The measurement of quality in ALCs is currently limited to state regulatory processes for compliance and safety, academic research studies, and any internal measurement that ALCs have the resources and competencies to perform themselves. However, quality measurement is complex and challenging, with dozens of quality measures and instruments compiled by the Center for Excellence in Assisted living and others, for ALCs to choose from.^13–15^ In addition, quality measurement is not sufficient if it is not combined with processes to compare quality to peer organizations and to systematically target quality improvement efforts based on the measurements and comparison. Some ALCs, owned by larger chains or members of provider associations, may have the resources and competency to implement internal processes to measure and improve quality, but with limited or no ability to compare themselves to a large number of peer ALCs.

Large-scale data-driven quality improvement collaboratives have improved quality across organizations in other health care sectors by enabling them to systematically measure, compare, and improve their quality.^16–18^ Having a system to collect and provide feedback on quality improvement variables is a critical aspect associated with organizational change and sustainability.^19^ It enables an organization to make data-driven quality improvement decisions. Audit and feedback are critical tools for introducing normative influences into the organizational work flow.^20–23^ Utilization of data-driven feedback reports represents a consistent component of quality improvement collaboratives.^24–26^ Comparison reports can be used to benchmark performance against peers and identify areas for improvement and then inform targeted support and adaptation to local contexts.^27^

Few initiatives in assisted living enable a large number of ALCs to measure, compare, or improve their quality, and almost none enable all 3 functions. A 2020 report to the state of Washington legislature listed existing initiatives in only 5 states—Wisconsin, New Jersey, Oregon, Ohio, and North Carolina—and 1 national initiative for members of the National Center for Assisted Living (NCAL) provider association.^28^ ALCs currently have limited options to engage in large-scale data-driven quality improvement by systematically measuring, comparing, and improving their quality. Systematic efforts to ensure quality are largely limited to quality assurance through compliance with state regulations. Although a few ALCs have the resources to engage in internal quality improvement, fewer have the resources to combine their quality improvement processes with quality measurement data systems. Overall, although the literature on quality in assisted living includes numerous measures and instruments to measure quality, little is known about the extent to which ALCs engage in quality measurement outside of research studies. Even less is known about the few existing initiatives that combine quality measurement, comparison, and quality improvement, and about how ALCs engage with them.

The objective of this article is to propose a model for large-scale data-driven quality improvement in assisted living and to evaluate the extent to which ALCs have implemented it between 2009 and 2021 in 1 state. Established definitions of implementation outcomes are considered in the evaluation: appropriateness, acceptability, adoption, feasibility, fidelity, penetration, and sustainability.^29^ The focus of this implementation study is the extent to which ALCs participate in large-scale data-driven quality improvement, rather than the extent to which large-scale data-driven quality improvement improves quality. The rationale of the study is that engaging ALCs proactively in quality measurement, comparison, and improvement is a worthwhile goal that complements and goes above and beyond the more reactive quality assurance through regulatory compliance.

## Methods

### Context

The proposed model for large-scale data-driven quality improvement in assisted living is based on 12 years of collaborative work in Wisconsin to provide a system and processes for ALCs to measure, compare, and improve their quality. To address the gap in bridging external quality assurance and internal quality improvement in assisted living, a public-private coalition was formed and sustained using a collaborative approach. This first implementation of the proposed model is called the Wisconsin Coalition for Collaborative Excellence in Assisted Living (WCCEAL). The WCCEAL coalition (wcceal.wisc.edu) was formed in 2009, and its quality improvement data infrastructure was codesigned by its advisory group in 2010–2012. ALCs have been implementing the WCCEAL model since 2013.

The proposed model of large-scale data-driven quality improvement emerged from the coalition’s work to align its public-private partners, informed by the key tenets of the Collective Impact framework from business and social innovation: cultivating a common agenda, shared measurement, mutually reinforcing activities, continuous communication, and backbone support.^30,31^

**Figure 1.**
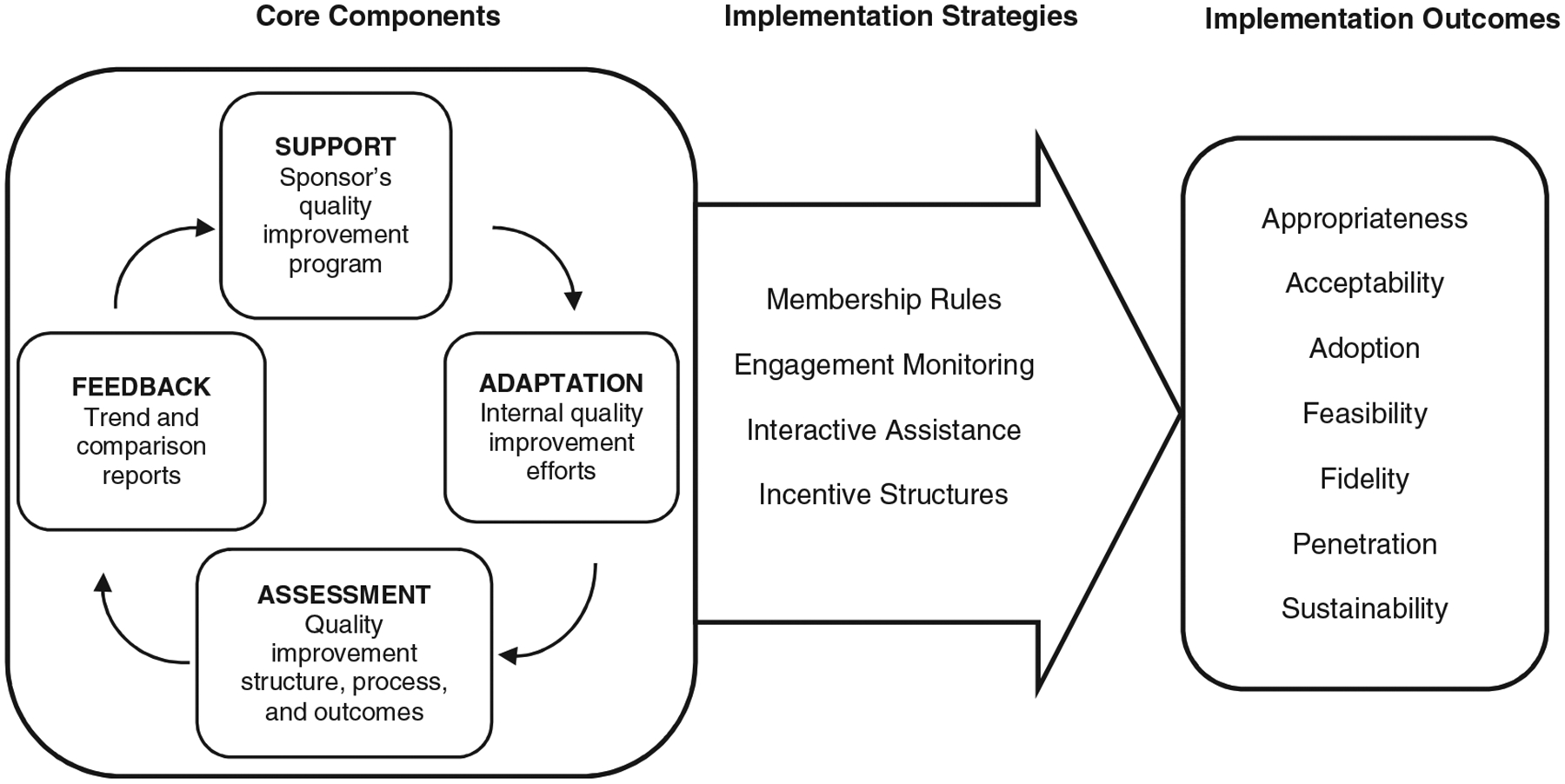
Implementing large-scale data-driven quality improvement. Proposed core components of large-scale data-driven quality improvement are assessment, feedback, support, and adaptation. Proposed strategies to implement these components are membership rules, engagement monitoring, interactive assistance, and incentive structures. Outcomes of using the strategies to implement the components are appropriateness, acceptability, adoption, feasibility, fidelity, penetration, and sustainability.

The coalition has continuously engaged in this alignment work since 2009 in monthly meetings of a multistakeholder advisory group. The advisory group includes representatives from the state regulatory and payer agencies, the 4 provider associations in the state, the state ombudsman program and resident advocates, and academic partners. The advisory group guides the specification, implementation, and continuous assessment and refinement of agreed on approaches designed to improve quality for assisted living residents. The advisory group uses consensus building communication to cultivate alignment among the partners. Through iterative stakeholder-driven codesign, the proposed model was developed and refined, including quality improvement intervention and implementation strategies (Figure 1).

### Intervention Core Components

The proposed model of large-scale data-driven quality improvement is a cyclical process consisting of 4 components: Assessment, Feedback, Support, and Adaptation (Table 1). Participating ALCs *assess* their quality improvement structure, processes, and outcomes (eg, falls, hospitalizations, infections, and challenging resident behaviors) by regularly self-reporting data at the ALC level (eg, quarterly) and administering surveys (eg, annual resident satisfaction). ALCs then review *feedback* reports that are automatically generated based on customized comparison groups they specify (eg, based on size, primary population, or license type) and aggregating data from all participating ALCs.^32^ This information then guides the targeted *support* that each ALC’s sponsoring organization (eg, provider association) provides to strengthen its quality improvement efforts.^24,25,33^ Finally, each ALC *adapts* how it endeavors to improve its quality to the specific requirements of its context and population, by evaluating its past and planning future efforts.

### Implementation Strategies

Four key implementation strategies^34^ have been specified to put the proposed model of large-scale data-driven quality improvement into practice (Table 2). They include *membership rules* outlining the requirements for ALCs to adopt and implement the model with fidelity, *engagement monitoring* to track participation over time and by member group, *interactive assistance* to help members with issues that may impede their implementation of the model, and *incentive structures* to reward member adoption and implementation of the model with fidelity.

### Implementation Study

#### Study design

In this implementation study, we conducted a mixed-methods evaluation of the application of the proposed model in Wisconsin, focusing on established implementation outcomes from implementation science,^29^ emphasizing the extent to which the model helped ALCs engage in measuring, comparing, and improving quality rather than whether ALCs’ quality improved. We used a parallel convergent mixed-methods design that is well suited to integrating qualitative data and quantitative data that are collected, separated, and then brought together to complement each other in addressing research questions of interest.

#### Measures

Table 3 describes how we evaluated implementation outcomes based on the established definitions of appropriateness, acceptability, adoption, feasibility, fidelity, penetration, and sustainability.^29^

#### Analysis

We used descriptive statistics for quantitative data on utilization of the web-based system, in relation to adoption, feasibility, fidelity, penetration (demographic), and sustainability. We compiled qualitative comments from stakeholders in relation to appropriateness, acceptability, and penetration (institutional).

## Results

### Appropriateness

At the inception of WCCEAL, a number of stakeholder groups within and outside the advisory group voiced concern that the WCCEAL approach and instruments appeared to be more compatible with the needs and constraints of ALCs serving older adults than other ALCs such as those serving individuals with developmental disabilities. Some ALCs also reported through their representatives on the advisory group that some parts of the quarterly instruments, such as assessment of falls with injury were not relevant to them because they had started with or achieved low falls rate. Beginning in 2015, participatory codesign stakeholder workgroups were formed to address the needs of WCCEAL ALCs serving multiple and diverse populations. The outcomes of these workgroups included the design and introduction of new questions on the quarterly data reporting, including questions related to challenging resident behaviors and medication errors. Based on advisory group meetings, the annual resident survey appears to be broadly relevant to all populations served by Wisconsin ALCs.

### Acceptability

All the stakeholders represented by the advisory group have described the quality improvement cycle implemented by WCCEAL as an acceptable intervention. Acceptability was assessed formatively through advisory group meetings over the course of the design, implementation, and operation of the main components of the intervention. Merely the collaborative establishment of the coalition and standardized measurement instruments is a major accomplishment in assisted living that is unprecedented and remains fairly unique in the country. The acceptability of the coalition is evidenced by the fact that public-private stakeholders have been participating in voluntary advisory group meetings every month since 2009. The acceptability of the standardized instruments is evidenced by the approval of the advisory group to deploy them to WCCEAL members, after being engaged in multiple design iterations and requests for feedback from broader constituents. This acceptability is further reinforced by the consistent use of the instruments by WCCEAL members.

### Adoption

Association membership is the main eligibility requirement for an ALC to enroll in WCCEAL. A total of 810 ALCs with unique state license numbers enrolled into WCCEAL between 2013 and the end of 2020. Of those, 256 disenrolled, and 91 may be a previously enrolled ALC with a new license number due to a change of ownership. As of the end of 2020, 487 ALCs were enrolled in WCCEAL, representing 31% of the 1573 ALCs in Wisconsin’s 4 assisted living associations and 11% of the total 4177 licensed ALCs in Wisconsin. The number of ALCs that are active members of WCCEAL has increased steadily over 33 quarters from 180 in the first quarter of 2013 to 506 in the first quarter of 2021.

Although the number of active WCCEAL members has grown and is higher than the number of ALCs in any known state or national initiative, the majority of licensed Wisconsin ALCs have not enrolled in WCCEAL. In 2021, to assess whether adoption can be increased without sponsor support by an association, 1-year Free Trial memberships of eQuality, WCCEAL’s online data and reporting system, were offered to any Wisconsin licensed ALC not currently in WCCEAL. Membership in an assisted living association was not a requirement for Free Trial participation, nor did the ALC receive association sponsorship during the Free Trial period. During the Free Trial, ALCs had access to the eQuality website to enter data and view reports, including quality improvement variables and resident satisfaction surveys. A total of 23 ALCs were enrolled in the Free Trial membership, following approval by the state regulatory agency. Through the first quarter of 2021, participation was limited, with only 17% submitting QI variables for the fourth quarter of 2020. The purpose of the Free Trial was to assess whether sponsor support by an association is an active ingredient for adoption and fidelity. Early results suggest that it is, thus, fulfilling the purpose of this assessment.

### Feasibility

Of all 810 ALCs with unique state license numbers that enrolled into WCCEAL since 2013, 746 (92%) completed at least 1 quarterly submission and 661 (82%) administered the annual survey at least 1 year, as shown in Table 4 along with the other implementation outcomes.

### Fidelity

ALC membership rules were designed and approved by the advisory group in 2015 and fidelity was assessed in terms of the proportions of WCCEAL member ALCs attaining the different levels of membership requirements. Between 2013 and 2020, the proportion of member ALCs completing the quarterly data submission was 88% on average, though it decreased steadily from 98% to 80% completion. Stakeholders believe this decrease was due first to the enduring workforce shortage, which was compounded by the pandemic last year. The annual proportion of member ALCs that administered the resident-level survey during the period 2013–2021 fluctuated in the 82% to 87% range, with a response rate fluctuating in the 49% to 57%. Of all 780 ALCs that were enrolled in WCCEAL for more than 2 quarters, 275 (35.3%) fulfilled their quarterly submission duties every eligible quarter and 338 (43.3%) fulfilled their survey submission duties during every eligible year.

As a secondary measure of fidelity, in the third quarter of 2019, Gold Member status was established, recognizing ALCs that go above and beyond the requirements of WCCEAL membership in any quarter. In addition to following all of the membership rules each quarter, Gold Members must also have been enrolled for more than 2 quarters and remain in good standing, must have submitted quality improvement variables on time, and must have viewed their new quality improvement variables reports by the end of the reports review period. They also must have reached a 25% return rate on the annual resident survey when applicable and must have viewed the resident survey reports by the end of the review period. In the third quarter of 2019, 68 (17%) of active ALCs achieved Gold Status, and in the second quarter of 2021, 165 (34%) of active ALCs achieved Gold Status.

### Penetration

In terms of demographic penetration, during 2013–2016, WCCEAL’s reach to Wisconsin residents increased steadily from 6024 to 10,883 licensed beds, increasing to 14,237 by the end of 2020, representing 23.8% of the 59,859 licensed beds in Wisconsin.

In addition, WCCEAL penetration in terms of institutionalization is evident among the stakeholder groups, which have allocated considerable new and existing staff resources to supporting WCCEAL, including it in the state and association annual budgets, and providing insurance premium discounts to WCCEAL members. Penetration at the national level is promising, in terms of institutionalization through federal matching funds from CMS and through increasing requests by other states for presentations about WCCEAL and what it would take to implement it.

WCCEAL’s penetration in terms of institutionalization is further evident in its trajectory from relying on research grant funding to becoming embedded into state government finances. Initial funding for development and implementation of the WCCEAL project was provided in 2011 by a 2-year pilot grant to build the infrastructure for the collaborative and in 2015 by a 5-year community impact grant. Since 2018, the eQuality system is now supported in the state annual budget along with a 25% yearly federal match. In 2019, WI DHS initiated an incentive program for Managed Care Organizations (MCOs) that provided increased funding for those MCOs with members in WCCEAL. Insurance companies in Wisconsin are recognizing the value of participation in a quality improvement program by providing discounts on liability insurance for WCCEAL member ALCs. Gold membership in WCCEAL has become a mark of commitment to quality in assisted living in Wisconsin, and WCCEAL is being recognized with multiple awards and honors to be a potential model for other states and nationally.

### Sustainability

Between 2013 and 2021, 810 facilities were enrolled in WCCEAL for an average of 15 quarters (nearly 4 years) with 506 member facilities as of 2021. Of the 746 ALCs that completed quality assessment for at least 1 quarter, 71% were enrolled for more than 2 years, with sustained early adopters representing 37% that have been enrolled for more than 5 years.

## Discussion

Building on supporting evidence from other health care sectors, we proposed a model for large-scale data-driven quality improvement in assisted living. We also provided evidence of low to moderate implementation outcomes of the model over the last decade. Although low to moderate, the implementation outcomes in the application of the model in Wisconsin are currently the highest in the assisted living sector for initiatives to support ALCs in measuring, comparing, and improving their quality. Although the rate of adoption is lower in Wisconsin than in the voluntary New Jersey initiative and the mandatory Oregon initiative, adoption in absolute terms is higher because Wisconsin has a much larger number of licensed ALCs in the state.

Based on the recent report to the state of Washington legislature,^28^ the initiatives in Wisconsin (formed in 2009), New Jersey (2012), and Oregon (2020) measure quality with ALC-level variables and resident-level surveys, whereas the initiative in Ohio collects resident and family surveys and the state of North Carolina provides a star rating as part of its regulatory inspection process. Participation is voluntary for ALCs in Wisconsin, New Jersey, and Ohio and mandatory for ALCs in Oregon, whereas North Carolina involves no participation outside of the regulatory process. Individual ALC results are reported publicly in Oregon, Ohio, and North Carolina. In Wisconsin, individual ALC results are reported internally to individual ALCs and their quality improvement sponsor (ie, provider association). Aggregate initiative-wide results are reported internally to all stakeholders in the initiative including state regulatory and payer agencies and the ombudsman program. Finally, ALCs participating in good standing are listed publicly in Wisconsin and ALCs meeting performance benchmarks are listed publicly with the designation of “advanced standing” in New Jersey. Nationally, NCAL provides a web-based tool for members to report quality metrics.

Of these initiatives, all but North Carolina provide ALCs support to measure quality, only Wisconsin, Oregon, and NCAL appear to provide ALCs support to compare their quality to other ALCs, and only Wisconsin, New Jersey, and NCAL appear to provide ALCs support to improve their quality in a way that is informed by quality measurement and comparisons, although New Jersey’s quality consulting appears to be focused on quality assurance and compliance.

Evaluation of a model like the one implemented in WCCEAL can focus on both long-term and short-term outcomes. In the long-term, longitudinal data enable the evaluation of the impact of an ALC being a member of WCCEAL on client outcomes (such as resident satisfaction) and service outcomes (such as safety and regulatory compliance). In the short term, the model was *appropriate* and *acceptable* to all the stakeholders and users, with steadily increasing *adoption* by Wisconsin ALCs, and continuing efforts to assess and improve its *appropriateness* for a broader range of ALCs and populations. Furthermore, participating in quality assessment has been *feasible* for most ALCs that adopted WCCEAL, the majority of which participate with *fidelity*, exceeding the membership requirements. WCCEAL’s *penetration* in Wisconsin is moderate in terms of the steadily increasing number of residents it reaches and high in terms of institutionalization in state budget and incentive structures.

The multistakeholder development of the proposed model and its implementation within WCCEAL is one of the few initiatives to enable ALCs to not only perform quality measurement but also tie it to large-scale comparisons and data-driven targeted improvement support. Its strengths include that it is not limited to 1 provider sponsor, drawing representation in its current implementation from all 4 major assisted living provider associations in Wisconsin, 3 of which are chapters of national associations. Its assessment also uses stakeholder-codesigned standardized questions that all members are required to use, rather than having ALCs select which questions to respond to and which to ignore. This allows for benchmarking across the entire coalition. Moreover, it uses different levels of data aggregation to facilitate active involvement of state government stakeholders without sharing the data of specific ALCs with the state regulators. This allows the communities to use WCCEAL to facilitate internal improvement with the support of their sponsors, comparing themselves freely to peer groups of their choice in user-customized reports. Finally, the response rates obtained thus far on ALC-level and resident-level data were high for voluntary surveys in this sector and population.

This study was limited to evaluating the implementation outcomes of the proposed model and did not evaluate the impact of implementing the model on ALC or resident outcomes. The focus on an implementation study rather than on an effectiveness study builds on a premise that has been demonstrated in other health care sectors, that engaging in systematic quality improvement informed by data-driven measurement and large-scale comparison is necessary to drive quality. An implementation study is appropriate in the presence of supporting evidence on effectiveness from other sectors. Future implementation-effectiveness hybrid studies^35^ are needed to confirm that the premise of effectiveness holds in the assisted living sector. A 2016 operational evaluation conducted internally based on WCCEAL data and state data did find that improvements in quality outcomes were higher in early adopters of the model than late adopters and non-adopters. However, research funding is needed to allow future studies to address selection threats to internal validity and confirm the effectiveness of the model for quality outcomes.

## Conclusions and Implications

Despite its demonstrated value in other health care sectors, large-scale data-driven quality improvement is still only minimally used in assisted living, where quality issues persist. We proposed a model for providing support for ALCs to measure quality and compare it to their peers to inform targeted improvement. The core components are *assessment* of structure, process, and outcomes, customizable *feedback* reports in comparison to self-selected peers, *support* from sponsoring organizations, and *adaptation* for targeted quality improvement efforts. Drawing on a decade of applying this model in a public-private collaborative in Wisconsin, we defined key implementation strategies for other collaboratives to tailor to local resources and constraints: specifying explicit *membership rules* with consequences, establishing processes for continuous *engagement monitoring* both quantitatively and qualitatively to provide targeted *interactive assistance* as needed, and integrating *incentive structures* into existing systems and policies where possible. This implementation study found that this model was highly *acceptable*, *appropriate*, and *feasible* for ALCs in Wisconsin. *Penetration* was high in terms of institutionalization into existing systems and policies, and moderate in terms of reach to nearly half of the residents of eligible ALCs. *Adoption* was moderate in the current implementation, and a recent pilot test suggested that support and interactive assistance from a sponsoring organization are active ingredients for adoption. *Fidelity* was high for membership requirements and current participation, and moderate for continuous participation. Sustainability was high in the medium term (2 years) and moderate in the long term (5 years) for both membership and participation. We hope this model and study will inform future work to evaluate and expand the few existing assisted living quality initiatives by complementing measurement with targeted comparisons and improvement support. More research is needed to study and address the challenges of wider implementation of large-scale data-driven quality improvement in assisted living as we reimagine the future of long-term care.

## Figures and Tables

**Table 1:** Core Components for Large-Scale Data-Driven Quality Improvement

Component	Description
Assessment	Participating ALCs assess their quality improvement structure, processes, and outcomes by self-reporting quarterly data and their residents’ quality of life and satisfaction by administering annual surveys. To complete the assessment, designated reporters in each ALC respond to a quarterly survey about quality improvement, including questions about structure (eg, staffing levels), process (eg, quality improvement activities), and outcome (eg, number of falls with injury). Additionally, ALCs administer an annual survey to their residents (eg, 28 items organized in 7 sections such as Staff, Environment, Health Management/Care), and answered on a 5-point Likert scale or “Not Applicable.”
Feedback	Written and graphical reports are provided to ALCs and their sponsors. Reports are available on demand and are updated each quarter with ALC data and annually with the data collected from resident surveys. ALCs and sponsors can customize the reports to include or exclude ALCs based on comparison filters such as care type, number of beds, and primary population served. Elements of the quarterly reports include bar graphs, box plots, trend reports, and tables, and like the annual satisfaction reports, they compare each ALC to all ALCs with the same sponsor and to all ALCs in the collaborative.
Support	Using the quality improvement information provided in the feedback stage, sponsor organizations provide targeted support to ALCs including coaching, networking, education, tools, and resources. The sponsors’ quality improvement programs provide ALCs with informational materials, training, and assistance to support their internal quality improvement efforts. Sponsor support may target ALC level to improve ALC structures, processes, and outcomes. An example is a workshop aimed at improving falls prevention programs. Sponsor support may also target resident satisfaction. An example of a resident-focused support intervention is inviting a national speaker to an annual conference to present best practices on meals and dining following low scores on that portion on the resident satisfaction survey. In addition to sponsor support, ALCs also receive support from the collaborative overall (eg free access to the Clinical Resource Center website (crc.wisc.edu), including AMDA best practice guidelines).
Adaptation	Building on the targeted support and the customized data reports, ALCs can take a data-driven approach to beginning or adjusting quality improvement efforts within their organization. ALCs can directly target suboptimal processes with improvement efforts. An example is an ALC continuously reducing its falls with injury by adapting its falls prevention programs based on trends and comparison reports along with coaching from its sponsor.

**Table 2:** Strategies to Implement Large-Scale Data-Driven Quality Improvement

Strategies	Descriptions
Membership rules	Membership rules include 2 criteria, Membership Conditions and Membership Duties. For example: Membership conditions are assessed by the state regulatory agency and the sponsoring organizations and include having a state assisted living license, membership in a provider association and the association’s quality improvement program, and no extreme regulatory action. Membership duties are assessed at the beginning of each quarter and include submitting quality improvement variables on time quarterly (at least 3 quarters in any 4 consecutive quarters) and submitting satisfaction surveys on time yearly, with at least 1 survey returned during the survey period. Satisfying all membership conditions and membership duties results in the ALC being listed on the public website of the collaborative, full access to the web-based password-protected information system, and regulatory flexibility if the ALC also qualifies for the state’s abbreviated survey process. Failure to satisfy membership duties or conditions results in the ALC moving into suspended or deactivated status and losing access to comparison reports. ALCs are expected to submit data while they work toward satisfying membership conditions and duties and moving back into listed member status.
Engagement monitoring	ALC engagement is primarily monitored through a web-based information system that consists of an annual resident satisfaction survey, an instrument to collect quality improvement variable data about each member ALC’s quality improvement outcomes, reports for ALCs and associations to monitor their data benchmarked against other participating ALCs, and quality improvement tools and resources for ALCs. Sponsoring organizations (eg, associations) have more extensive information available to them on individual ALC participation and performance. Engagement is also monitored at monthly advisory group meetings.
Interactive assistance	ALCs receive assistance from sponsors and the collaborative as a whole to adopt and implement the model with fidelity. ALCs receive interactive assistance from sponsoring organizations (eg associations) in addition to the support they receive through participation in sponsoring organizations’ quality improvement programs. A helpdesk also provides interactive assistance and user access support to ALCs and associations.
Incentive structures	Incentives are provided by various stakeholder groups to reward ALCs adopting and implementing the model with fidelity. Examples for ALCs that are members in good standing include Regulatory flexibility: ALCs that qualify for the state regulatory agency’s abbreviated surveys that are members in good standing are surveyed later than other ALCs. Recognition on the collaborative’s public website. Eligibility for discounts on liability insurance. Additional funding and support from managed care organizations (MCOs) offering performance-based incentives.

**Table 3:** Implementation Outcomes Measurement

Implementation Outcomes	Measurement
Appropriateness	Appropriateness (perceived fit) of the intervention components and the implementation strategies was based on the perception of stakeholders as represented by the advisory group.
Acceptability	Acceptability was assessed formatively through advisory group meetings over the course of the design, implementation, and operation of the main components of the innovation.
Adoption	Adoption was measured by the number of ALCs ever and currently actively enrolled, and the proportion of eligible potential adopters that they represent. Membership in one of the 4 assisted living associations’ quality improvement programs is the main eligibility requirement.
Feasibility	Feasibility (actual fit) was measured by the number of enrolled ALCs that were able to complete at least 1 quarterly quality assessment or administer the annual resident survey at least once.
Fidelity	Fidelity of implementation of the intervention was informed by ALC membership rules that were designed and approved by the advisory group in 2015. Fidelity was assessed in terms of the proportions of member ALCs meeting or exceeding the membership requirements.
Penetration	Penetration was defined in 2 ways, institutionally and demographically. Institutionally, it was defined in terms of the integration of the model into existing structures and systems. Demographically, it was defined in terms of the proportion of eligible residents reached.
Sustainability	Sustainability was defined by continued ALC participation in, and further expansion of, the system and coalition. It was measured by the number of ALCs enrolled or completed quality assessment for more than a certain number of quarters.

**Table 4:** Implementation Outcomes for Large-Scale Data-Driven Quality Improvement in Assisted Living in Wisconsin

Implementation Outcomes	n	%
Adoption		
Total enrollments between 2013 and 2020	810	n/a
Members enrolled as of end of 2020 (n = 1573 eligible)*	487	(31)
Feasibility		
Members that completed quality assessment ≥1 quarter (n = 810)^†^	746	(92)
Members that administered resident survey ≥1 y (n = 810)^†^	661	(82)
Fidelity		
Membership		
Members never suspended from membership in good standing (n = 780 eligible)	538	(69)
Average members in good standing per quarter^‡^	n/a	(88)
Participation		
Members that completed quality assessment in Q1 2021 (n = 506 eligible)	407	(80)
Continuous participation		
Members that never missed a quarterly quality assessment (n = 780 eligible)^§^	275	(35)
Members that never missed an annual resident survey (n = 780 eligible)^§^	338	(43)
Penetration		
Residents in member communities as of end of 2020^||^ (n = 30,345 eligible**)	14,237	(47)
Sustainability		
Membership		
Members enrolled >2 y (n = 746)	529	(71)
Members enrolled >5 y (n = 746)	277	(37)
Participation		
Members that completed quality assessment >8 quarters (n = 746)	468	(63)
Members that completed quality assessment >20 quarters (n = 746)	230	(31)

n/a: not available due to variable n by quarter.

* Belong to one of the 4 Wisconsin provider associations.

† Member enrollments since 2013.

‡ Quarterly average since definition of membership rules in Q2 2015.

§ Enrolled ≥2 quarters.

|| Estimated as number of licensed beds in enrolled communities.

** Licensed beds in communities belonging to one of the 4 WI provider associations.
